# Comprehensive analyses for the coagulation and macrophage-related genes to reveal their joint roles in the prognosis and immunotherapy of lung adenocarcinoma patients

**DOI:** 10.3389/fimmu.2023.1273422

**Published:** 2023-10-31

**Authors:** Zhuoqi Li, Zongxiu Yin, Zupeng Luan, Chi Zhang, Yuanyuan Wang, Kai Zhang, Feng Chen, Zhensong Yang, Yuan Tian

**Affiliations:** ^1^ Department of Otolaryngology-Head and Neck Surgery, Shandong Provincial ENT Hospital, Shandong University, Jinan, China; ^2^ Radiotherapy Department, Shandong Second Provincial General Hospital, Shandong University, Jinan, China; ^3^ Department of Pulmonary and Critical Care Medicine, Jinan Central Hospital Affiliated to Shandong First Medical University, Jinan, China; ^4^ Department of Radiation Oncology, Jinan Third People’s Hospital, Jinan, China; ^5^ Department of Cardiology, The Second Hospital, Cheeloo College of Medicine, Shandong University, Jinan, China; ^6^ Department of Oncology, The Second Affiliated Hospital of Shandong University of Traditional Chinese Medicine, Jinan, China; ^7^ Generalsurgery Department, Wen-shang County People’s Hospital, Wenshang, China; ^8^ Department of Thoracic Surgery, Shandong Cancer Hospital and Institute, Shandong First Medical University and Shandong Academy of Medical Sciences, Jinan, China; ^9^ Department of Gastrointestinal Surgery, Yantai Yuhuangding Hospital, Qingdao University, Yantai, China

**Keywords:** lung adenocarcinoma, coagulation, tumor-associated macrophage, risk score model, prognosis, immunotherapy

## Abstract

**Purpose:**

This study aims to explore novel biomarkers related to the coagulation process and tumor-associated macrophage (TAM) infiltration in lung adenocarcinoma (LUAD).

**Methods:**

The macrophage M2-related genes were obtained by Weighted Gene Co-expression Network Analysis (WGCNA) in bulk RNA-seq data, while the TAM marker genes were identified by analyzing the scRNA-seq data, and the coagulation-associated genes were obtained from MSigDB and KEGG databases. Survival analysis was performed for the intersectional genes. A risk score model was subsequently constructed based on the survival-related genes for prognosis prediction and validated in external datasets.

**Results:**

In total, 33 coagulation and macrophage-related (COMAR) genes were obtained, 19 of which were selected for the risk score model construction. Finally, 10 survival-associated genes (APOE, ARRB2, C1QB, F13A1, FCGR2A, FYN, ITGB2, MMP9, OLR1, and VSIG4) were involved in the COMAR risk score model. According to the risk score, patients were equally divided into low- and high-risk groups, and the prognosis of patients in the high-risk group was significantly worse than that in the low-risk group. The ROC curve indicated that the risk score model had high sensitivity and specificity, which was validated in multiple external datasets. Moreover, the model also had high efficacy in predicting the clinical outcomes of LUAD patients who received anti-PD-1/PD-L1 immunotherapy.

**Conclusion:**

The COMAR risk score model constructed in this study has excellent predictive value for the prognosis and immunotherapeutic clinical outcomes of patients with LUAD, which provides potential biomarkers for the treatment and prognostic prediction.

## 1 Introduction

Although the screening and treatment of lung cancer have witnessed greater improvement in the past few years, there are still ongoing challenges in improving the clinical outcomes of patients (1, 2). Lung adenocarcinoma (LUAD), a kind of non-small cell lung cancer (NSCLC), was the most common lung malignancy with genetic and morphologic diversity, and the pathogenesis and treatment of LUAD still need further exploration (3, 4). The tumor microenvironment (TME) plays a critical role in tumor progression and treatment (5, 6). Tumor-associated macrophage (TAM) was an essential component of the tumor microenvironment, and it contributed to tumor growth, metastasis, and immunosuppression, as well as tumor resistance to chemotherapy and checkpoint blockade immunotherapy (7, 8).

There were also a number of studies about the roles of TAMs in NSCLC or LUAD. TAMs in the TME usually originated from two main sources: one was the bone marrow (BM)-derived monocytic precursors; another was the tissue-resident macrophages (TRMs) originated from embryonic precursors (8). After egress from the BM, monocytes (or M-MDSCs) were recruited to the TME via chemokines of the CC and CXC families, such as CCL2, CCL5, and CXCL12, that were produced by cancer cells early during tumorigenesis (9). Subsequently, the myeloid cells recruited to tumors would convert to TAMs under the activation of integrin (9). CCR2 and CX3CR1 were the receptors of the chemokines CCL2 and CX3CL1, respectively, and they were proven to play significant roles in macrophage migrating to lung cancer and M2 polarization (10).

TAMs shaped the TME of NSCLC. They accumulated close to tumor cells in the early stage of tumor formation to promote epithelial–mesenchymal transition and invasiveness of tumor cells, and they also caused a potent regulatory T-cell response that suppressed the adaptive immunity of tumor cells (11). TAMs can promote LUAD growth or metastasis by secreting some factors that can be adopted by the tumor cells in the TME, such as miR-942 (12), LINC00273 (13), and HB-EGF (14), as well as by upregulating CRYAB expression in tumor cells (15). The M2 subtype of TAM enhances the expression of VEGF-A and VEGF-C, which is significantly associated with angiogenesis and lymphangiogenesis, contributing to the progression of NSCLC (16). TAMs also have a great impact on the chemotherapy and anti-PD1/PD-L1 immunotherapy for LUAD (17, 18). Recent studies found that TAMs had a close relationship with coagulation. On the one hand, TAM was an important contributor to the coagulation in tumors by producing factor X (FX) and leading to cell-autonomous FXa-PAR2 signaling in these cells within the TME (19, 20). On the other hand, some coagulation-related factors can regulate the functions of TAMs, consequently influencing the progression of tumors. For example, thrombin and plasminogen activator inhibitor-1 (PAI-1) can facilitate the M2 polarization of TAMs in ovarian and breast cancer, respectively (21, 22). Tissue factor (TF) expression by tumor cells can recruit TAMs to the lung, supporting the formation of the premetastatic niche (23). The lung plays an important role in blood coagulation, and there was evidence that the lung is a primary site of terminal platelet production (24). Lung cancer is a non-negligible cause of the disturbance of blood coagulation, which can lead to venous thromboembolism, the second leading cause of death in cancer patients (5, 25). NSCLC has a relatively high risk of venous thromboembolism among lung cancer types, and LUAD is especially an independent risk factor for it (26, 27). The pathophysiology of this phenomenon was complex and not entirely understood, and several related risk factors were involved (25).

The study was designed to further explore the significance of coagulation and TAM infiltration in shaping the TME of LUAD and predicting the prognosis and immunotherapeutic clinical outcomes of LUAD patients.

## 2 Materials and methods

### 2.1 Data collection and preprocessing

The gene expression profiles of The Cancer Genome Atlas (TCGA)-LUAD cohort (converted to log2(FPKM+1)) were downloaded using the R package “TCGAbiolinks”. The officially corrected survival information (overall survival (OS)) and clinical information (including age, stage, gender, grade, etc.) of LUAD patients in TCGA were downloaded from the cBioPortal database.

The gene expression profiles and clinical information of the GSE30219, GSE37745, GSE41271, GSE42127, GSE50081, GSE68465, and GSE72094 datasets were downloaded from the GEO database. In these datasets, the primary tumors were collected by surgical resection from lung adenocarcinoma patients. The patients in these cohorts have been collected with high-quality gene expression data and complete clinical and follow-up information. None of the patients received preoperative chemotherapy or radiotherapy. The probes in the GEO datasets corresponding to more than one gene would be removed. When multiple probes corresponded to the same symbol, the average value would be taken.

We filtered out the samples with incomplete survival information in TCGA and the GEO datasets. The GSE68465 dataset was used as the training cohort, while the other datasets were taken as the validation cohorts. The GSE131907 dataset, containing single-cell transcriptome data from 15 lung adenocarcinoma patients, was also downloaded from the GEO database. The cellular annotation results, reported by Kim, were used for the subsequent analyses (28). A total of 535 coagulation-related genes were obtained from the coagulation-related pathways in the MSigDB and Kyoto Encyclopedia of Genes and Genomes (KEGG)databases. The detailed pathways and the numbers of the corresponding genes were listed in (**Table 1**), and the names of those 535 genes are listed in **Supplementary Table S1**.

**Table 1:** The coagulation-related pathways and the number of genes involved in each pathway.

Pathways	Count
GOBP_BLOOD_COAGULATION_INTRINSIC_PATHWAY	18
GOBP_COAGULATION	347
GOBP_NEGATIVE_REGULATION_OF_COAGULATION	52
GOBP_POSITIVE_REGULATION_OF_COAGULATION	24
GOBP_REGULATION_OF_COAGULATION	71
HALLMARK_COAGULATION	138
KEGG_COMPLEMENT_AND_COAGULATION_CASCADES	69
KEGG_PLATELET_ACTIVATION	124
KEGG_COMPLEMENT_AND_COAGULATION_CASCADES	86

### 2.2 The construction of the gene co-expression network by WGCNA analysis

Weighted Gene Co-expression Network Analysis (WGCNA) aimed to identify co-expressed gene modules, explore the relationships between the gene co-expression networks and the phenotypes of interest, and study the core genes in the network. WGCNA analysis was performed using the genes with the top 75% highest variation coefficient in the expression profile of the GSE68465 dataset. First, the correlation coefficient between every two genes was calculated, and the connections between genes in the network were made to obey a scale-free network using the weighted values of the correlation coefficients. Subsequently, a hierarchical clustering tree was constructed based on the correlation coefficients among these genes. Different branches of the clustering tree represented different gene modules, and different colors represented different modules. Next, the significance of the modules was calculated and used to calculate the correlation between the macrophage M2 infiltration scores and different modules, and the genes in each module, considered signature genes of the modules, were recorded.

### 2.3 Processing the single-cell RNA-seq data

The R package “Seurat” was used to preprocess the scRNA-seq data. First, we set the following thresholds in which the cells can be included in the study: (1) cells with more than 200 and less than 10,000 genes; (2) cells with less than 20% mitochondrial gene expression; and (3) cells with more than 100 and less than 150,000 UMIs. The “NormalizeData” function was used to normalize the scRNA-seq dataset, and 3,000 highly variable genes were identified using the “mvp” method of the “FindVariableFeatures” function. Subsequently, we made scale transformed for the data and performed principal component analysis (PCA) for dimensionality reduction. We eventually selected the top 20 principal components for the downstream analyses. Since the data were obtained from different samples, batch correction was performed using the R package “Harmony” to avoid the interference of the batch effect on the subsequent analyses. We used the UMAP algorithm to mine and visualize the data. Finally, we annotated the cell populations based on the signatures provided by the study of Kim et al. (28).

We identified differentially expressed genes (DEGs) between each cell type by using the “FindAllMarkers” function in the R package “Seurat”, where min.pct = 0.1, logfc. threshold = 0.25, and only.pos = FALSE were set, while only genes with *p*-values of< 0.05 would be retained. We used the R package “scRNAtoolVis” to plot the volcano chart for the DEGs between different cell types.

### 2.4 Mutation and CNV analyses

R package “maftools” was employed to plot the waterfall maps of the mutation landscape of the 33 coagulation and macrophage-related (COMAR) genes in the TCGA-LUAD cohort. The CNV data of TCGA-LUAD was downloaded from the “UCSC Xena” website, and then the CNV frequency was presented in a plot finished by R software.

### 2.5 Construction of the COMAR prognostic model

The COMAR prognostic model was constructed based on 33 coagulation and macrophage-related genes. First, Kaplan-Meier survival analysis was performed to divide the patients into high and low-expression groups with the best cut-off value for each gene, and 19 genes that had significant differences in survival status between the two groups were identified. Next, multivariate Cox regression analysis for the 19 genes was used to construct the 10-gene prognostic model. In the COMAR prognostic model, patients’ risk scores were calculated based on the expression levels of each prognosis-related gene and their corresponding regression coefficients:


$$\text{Risk score}=\sum_{i=1}^{n}\text{expi}*\;\beta \text{i}$$

In the above formula, “*n*” represents the number of genes; “expi” represents the expression level of gene “i”; and “βi” represents the coefficient of gene “i”. Patients were divided into high- and low-risk groups according to the median risk score, and survival analysis was performed using the R package “survminer” to analyze OS in the high- and low-risk groups. The “survminer” and “timeROC” packages were used to perform time-dependent ROC curve analysis to check the predictive efficacy of the prognostic models. Finally, risk scores would be calculated in the validation cohorts using the same formula.

### 2.6 Biological functional annotation

The GO_BP and GO_MF enrichment analyses were performed using the Gene Set Variation Analysis (GSVA) algorithm to calculate the score for each pathway in each sample. The differentially activated pathways in the high- and low-risk score groups were identified using the “limma” package, with the differential threshold set at FDR< 0.05. Differentially activated KEGG pathways between the high- and low-risk score groups were analyzed using Gene Set Enrichment Analysis (GSEA).

### 2.7 The estimation of immune cell infiltration in the TME

The CIBERSORT algorithm in the R package “IOBR” was applied to evaluate the immune cell abundance in the samples of the GSE68465 dataset. Specifically, the CIBERSORT algorithm was used to calculate the infiltration fractions of the 22 types of immune cells. CIBERSORT was considered superior to previous methods of deconvolution when analyzing unknown mixture content and noise. This algorithm could be used to statistically estimate the relative proportions of cell subgroups in complex tissues according to gene expression profiles, making it a useful tool for estimating the abundance of specific cell types in mixed tissues.

### 2.8 Collecting the immunotherapeutic cohorts

The GSE126044 dataset, containing seven LUAD patients who received anti-PD-1 immunotherapy, was downloaded from the GEO database. The GSE135222 dataset containing 27 NSCLC patients with anti-PD1/PD-L1 immunotherapy was also downloaded from the GEO database. We calculated the risk scores for each sample in these datasets using the same algorithm as the previous model and made a survival analysis. We also compared the difference in risk score between the patients with cancer progression and those with no progression after receiving immunotherapy.

### 2.9 Statistical analysis

All the analyses were performed in R software (version 4.1.2). For significance analysis between various values (such as expression levels, infiltration ratio, and various eigenvalues, etc.), the Wilcoxon rank-sum test was applied to compare the differences between two groups of samples, while the Kruskal–Wallis test was used to compare the differences between multiple groups of samples. For plot presentation, the “ns” represents p > 0.05; “*” represents p< 0.05; “**” represents p< 0.01; “***” represents p< 0.001; and “****” represents p< 0.0001. Survival curves in the prognostic analysis were generated by the Kaplan–Meier method, and the significance of the differences was determined by the log-rank test.

## 3 Results

### 3.1 Screening the macrophage-related genes through WGCNA

**Figure 1:**
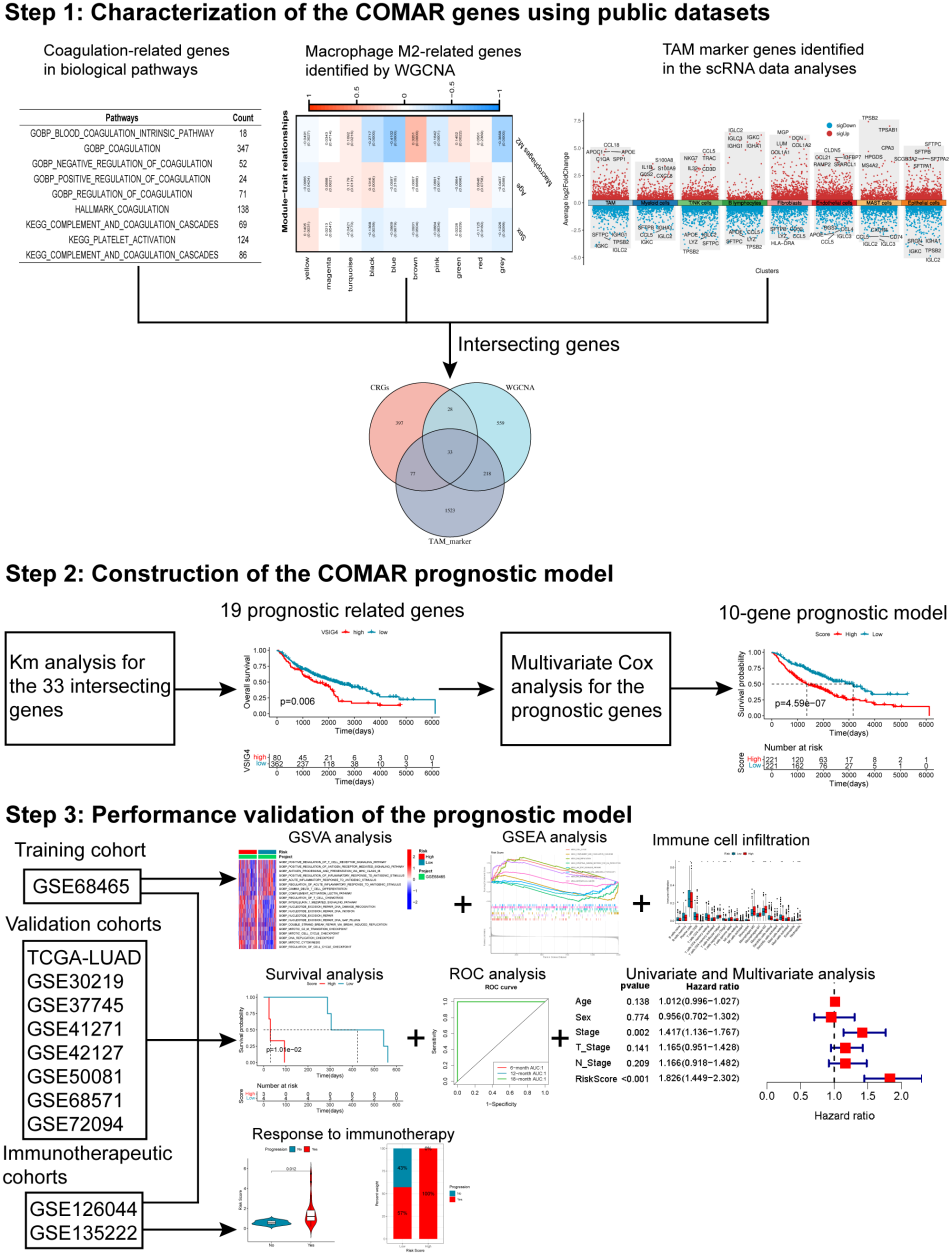
The flow chart of this study.

The flow chart of this study is shown in **Figure 1**. The CIBERSORT algorithm was used to calculate the content of macrophages M1 and M2 in the samples of the GSE68465 cohort. Next, the LUAD patients were divided into groups with high and low macrophages M1 and M2. Kaplan–Meier analysis indicated that there was no significant difference in the survival of LUAD patients between the high and low macrophage M1 groups (**Supplementary Figure S1A**), but patients in the low macrophage M2 group had a longer overall survival (**Supplementary Figure S1B**). This suggested that macrophage M2 played an important role in LUAD. Based on this result, WGCNA was used to identify macrophage M2-related genes in LUAD. First, the result of sample clustering showed no outliers in these LUAD samples (**Supplementary Figure S1C**). When the power value was 7, the degree of independence was > 0.85 for the first time, so 7 was selected as the optimal soft threshold power (**Supplementary Figures S1D**, **S2E**). There were nine gene modules identified in the WGCNA (**Supplementary Figures S1F**, **S2G**). The correlation analysis indicated that genes in the brown module (cor = 0.33, *p* = 0.0001) and blue module (cor = −0.41, *p* = 0.0000) were most significantly correlated with macrophages M2. Therefore, 408 genes in the brown module and 430 genes in the blue module (**Supplementary Table S2**) were selected for the subsequent analyses.

### 3.2 Acquiring the TAM marker genes using scRNA-seq data

After quality control for the scRNA-seq dataset GSE131907, 25,011 genes were detected in 50,515 cells. The violin plots showed the number of genes detected in each cell (nFeature), the total number of counts in each cell (nCount), and the percentage of mitochondrial genes in each cell (percent.mt) (**Supplementary Figures S2A–C**). The correlation analysis indicated that nCount was significantly positively correlated with nFeature (**Supplementary Figure S2D**). Next, the 3,000 highly variable genes were plotted in the scatter plot (**Supplementary Figure S2E**). In total, 20 PCs were identified by PCA (**Supplementary Figure S2F**), which were selected for “harmony” analysis. According to the TSNE and cell type annotation, all cells were divided into two groups (34,279 immune cells and 16,236 nonimmune cells). The immune cell group consisted of B lymphocytes, mast cells, myeloid cells, T/NK cells, and TAM, while the nonimmune cell group included endothelial cells, epithelial cells, and fibroblasts (**Supplementary Figures S3A**, **S4B**). Differentially expressed genes for each cell type were analyzed and displayed in the volcano plot (**Supplementary Figure S3C**). The 1,815 differentially expressed genes in TAM were considered the TAM-associated genes (**Supplementary Table S3**).

### 3.3 Characterization of the COMAR genes and the landscape of their genetic and transcriptional alterations

**Figure 2:**
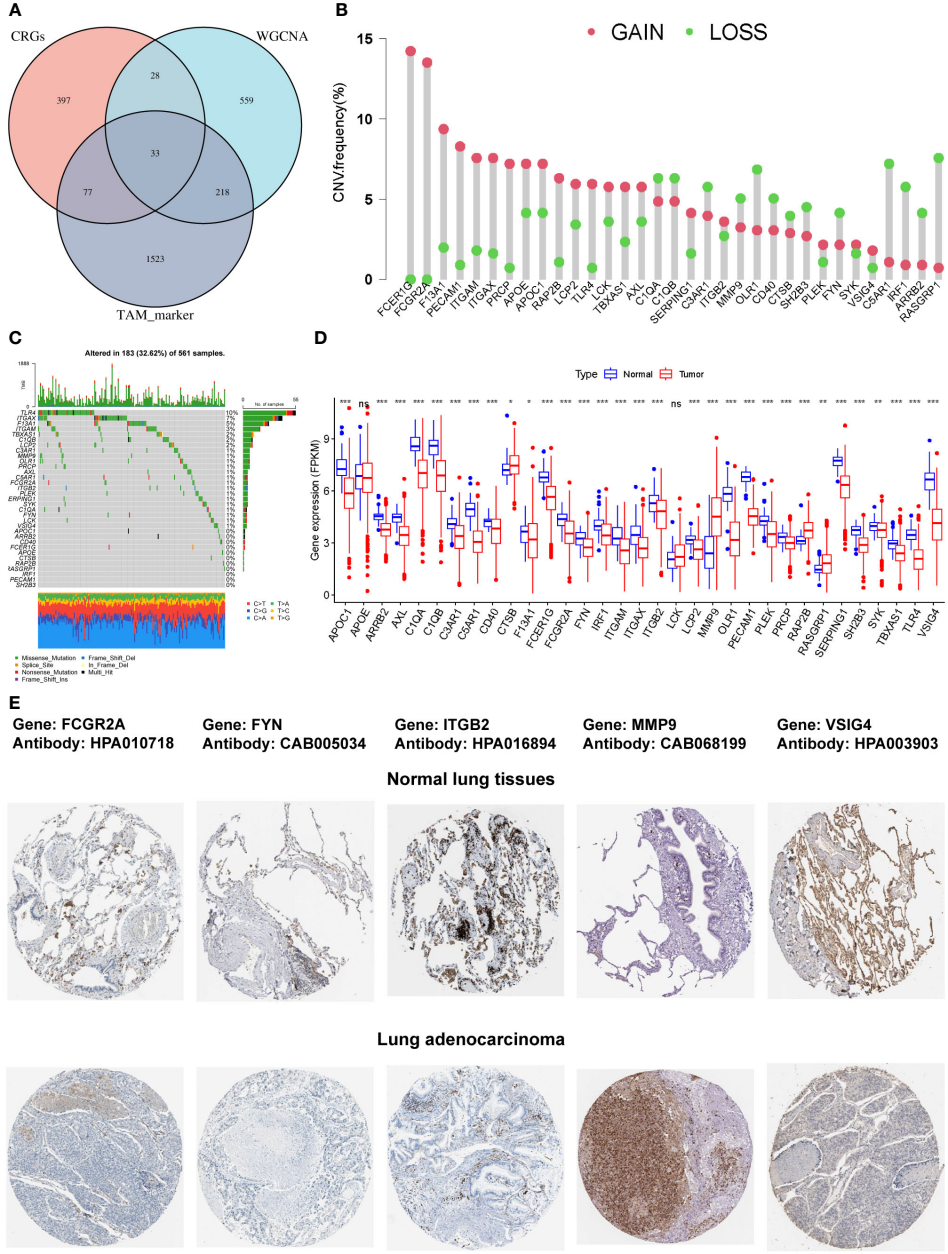
The characterization of the 33 COMAR genes and the landscape of their genetic and transcriptional alterations. **(A)** The determination of the 33 COMAR genes from the cross-talk of the coagulation-related genes, the macrophage M2-related genes identified by WGCNA, and the TAM markers. **(B)** The mutational frequency of the 33 coagulation-associated genes in 561 LUAD patients from the TCGA-LUAD cohort. Each column represents individual patients. Upper bar plots show TMB, and the numbers on the right indicate the mutational frequency of each gene. Right-bar plots show the proportion of each variant type. Stacked bar plots below show the fraction of conversions in each sample. **(C)** The CNV variation frequency of the 33 coagulation-related genes in the TCGA-LUAD cohort. The height of the column represents the alteration frequency. Red dots represent deletion frequency; blue dots represent amplification frequency. **(D)** The expression levels of the 33 genes between normal and LUAD cancer tissues in the TCGA-LUAD cohort. In the box plot, blue represents normal tissues, and red represents cancer tissue. The upper and lower ends of the boxes represent the interquartile ranges of values. Lines in the boxes represent median values. Blue or red dots show outliers. Asterisks above the boxes represent the *p*-value (^*^ *p*< 0.05; ^**^ *p*< 0.01; ^***^ *p*< 0.001; ns, p> 0.05). **(E)** The immunohistochemical staining images of FCGR2A, FYN, ITGB2, MMP9, and VSIG4 genes in normal lung tissues and LUAD tumor tissues. The names of genes and antibodies are listed at the top of the figure. The upper five images are the staining in the corresponding normal tissues, and the lower five images are the staining in the tumor tissues.

The intersection of the 535 coagulation-associated genes, 838 macrophage M2-related genes, and 1,815 TAM-associated genes contained 33 genes, and these genes were selected for the subsequent analyses (**Figure 2A**; **Supplementary Table S4**). We first summarized the incidence of copy number variations and somatic mutations of the 33 COMAR genes in LUAD. Among 561 samples, 183 experienced mutations of coagulation-related genes, with a frequency of 32.62%. It was found that the TLR4 exhibited the highest mutation frequency, followed by ITGAX, while 11 genes did not show any mutations in LUAD samples (**Figure 2C**). The investigation of CNV alteration frequency indicated a prevalent CNV alteration in these coagulation-related genes, with copy number amplification being much more significant than copy number deletion. Genes like FCER1G and FCGR2A were found with pretty prominent copy number amplification, while RASGRP1 and C5AR1 were found with obvious copy number deletion (**Figure 2B**). We also compared the relative RNA expression levels between LUAD and paired normal tissues and found that most of the genes were downregulated in LUAD compared with paired normal tissues (**Figure 2D**). Thus, there may be some other factors that may influence the expression of these genes, except for CNV. The Human Protein Atlas (HPA) database was applied to validate the protein expression of the COMAR genes, and the IHC staining images of FCGR2A, FYN, ITGB2, MMP9, and VSIG4 were obtained (**Figure 2E**). Each gene was stained using the same antibody in the normal lung tissue and LUAD cancer tissue. Among these genes, FCGR2A, FYN, ITGB2, and VSIG4 protein levels were increased in tumor tissues, while MMP9 protein level was decreased, which was consistent with their mRNA expression levels (**Figures 2D, E**).

### 3.4 Construction and validation of the prognostic model based on the COMAR genes

**Figure 3:**
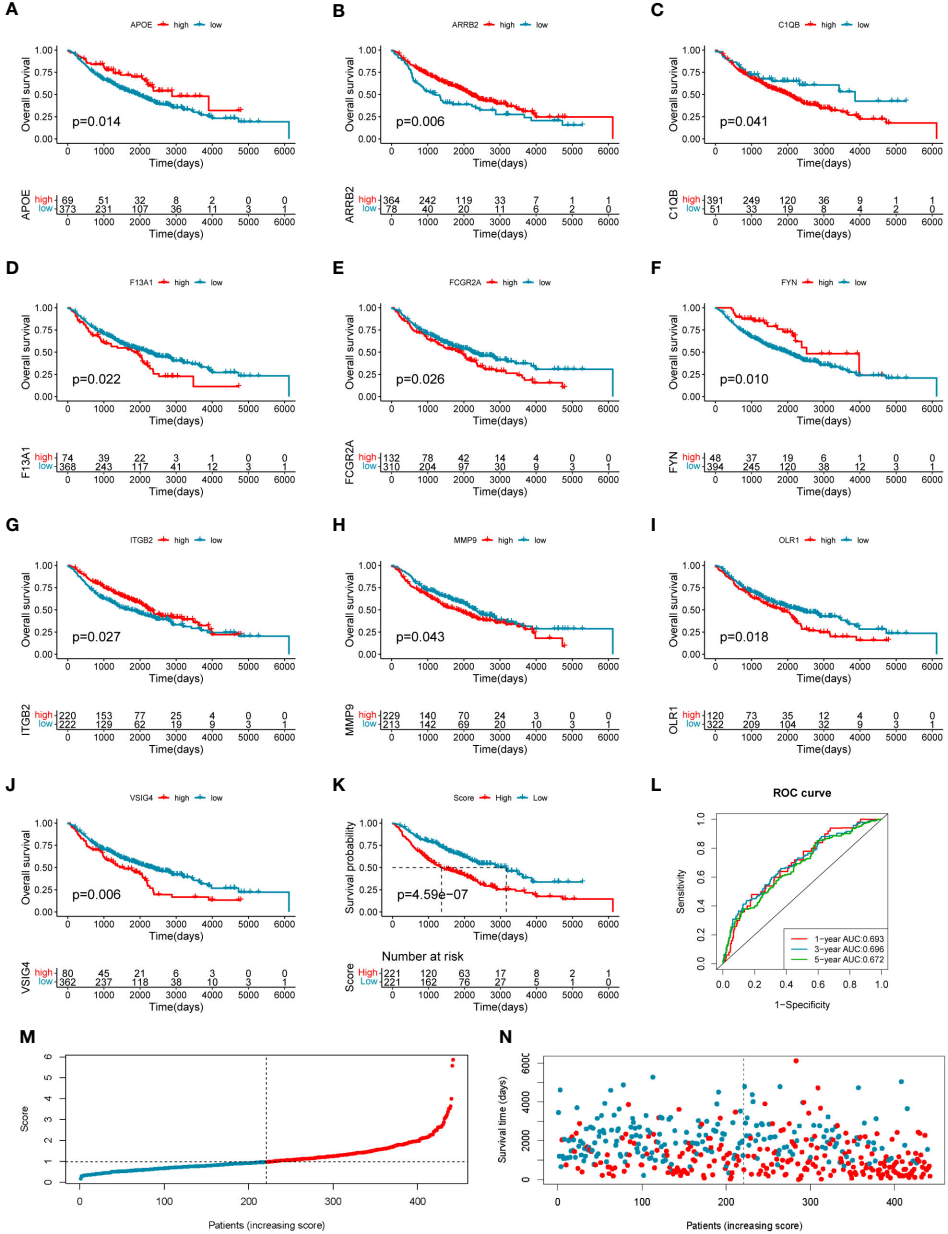
Construction of the 10-gene prognostic model in the training cohort. **(A–J)** The overall survival curves of the 10 genes involved in the prognostic model: **(A)** APOE, **(B)** ARRB2, **(C)** C1QB, **(D)** F13A1, **(E)** FCGR2A, **(F)** FYN, **(G)** ITGB2, **(H)** MMP9, **(I)** OLR1, and **(J)** VSIG4. The abscissa axis shows survival time, while the ordinate axis shows survival probability. Blue represents low expression, while red represents high expression. The grouping status of the patients is indicated at the bottom of the chart. *p*< 0.05 in the Log-rank test was considered statistically significant. **(K)** The overall survival curve of patients in high- and low-risk score groups in the training cohort. The abscissa axis shows survival time, while the ordinate axis shows survival probability. Blue represents patients with low-risk scores, while red represents patients with high-risk scores. The grouping status of the patients is indicated at the bottom of the chart. P< 0.05 in the Log-rank test was considered statistically significant. **(L)** The ROC curve for predicting the 1-, 3-, and 5-year survival of LUAD patients according to the risk score. The abscissa axis represents specificity and the vertical axis represents sensitivity. Different colors represent different predictive times. **(M)** The risk score distributions of the patients. **(N)** The survival status of the patients.

To investigate the clinical value of the 33 COMAR genes, we divided the patients in the training cohort GSE68465 into high- and low-expression groups for each gene with the best cut-off value and performed survival analysis. Results indicated that 19 genes were prognostic-related genes (**Supplementary Figure S4**). We then conducted a multivariate Cox regression analysis based on the 19 genes. Finally, 10 of the 19 genes were found in the prognostic model we constructed (**Figures 3A–J**). The specific calculation formula for the risk score model was listed as follows:

Risk score = (−0.26708659 * APOE expression level) + (−0.282614466 * ARRB2 expression level) + (0.410059345 * C1QB expression level) + (0.178659465 * F13A1 expression level) + (−0.303985307 * FCGR2A expression level) + (−0.271534215 * FYN expression level) + (−0.610784492 * ITGB2 expression level) + (0.15191577 * MMP9 expression level) + (0.120339218 * OLR1 expression level) + (0.446920184 * VSIG4 expression level).

The training LUAD patients were ranked by the risk score and divided into low-risk (*n* = 221) and high-risk (*n* = 221) groups (**Figure 3M**; **Supplementary Table S5**), and the patient’s survival time became shorter with the risk score increasing generally (**Figure 3N**). The Kaplan–Meier curve showed a significantly poorer prognosis in the high-risk group than in the low-risk group (log-rank test, *p* = 4.59*e*−07) (**Figure 3K**). The ROC curve showed the AUCs of the patients at 1, 3, and 5 years were 0.693, 0.696, and 0.672, respectively (**Figure 3L**). The AUCs in the prediction of short-term prognosis were higher, and they were 0.745, 0.740, and 0.718 at 4-, 6-, and 9-month follow-up, respectively (**Supplementary Figure S5A**). Thus, the prognostic model might have stronger predictive efficacy for shorter-term prognosis. Moreover, this prognostic model had significantly superior predictive efficacy compared with other clinical factors such as age, sex, tumor stage, and differentiation status at 1-, 3-, and 5-year follow-ups (**Supplementary Figures S5B–D**).

**Figure 4:**
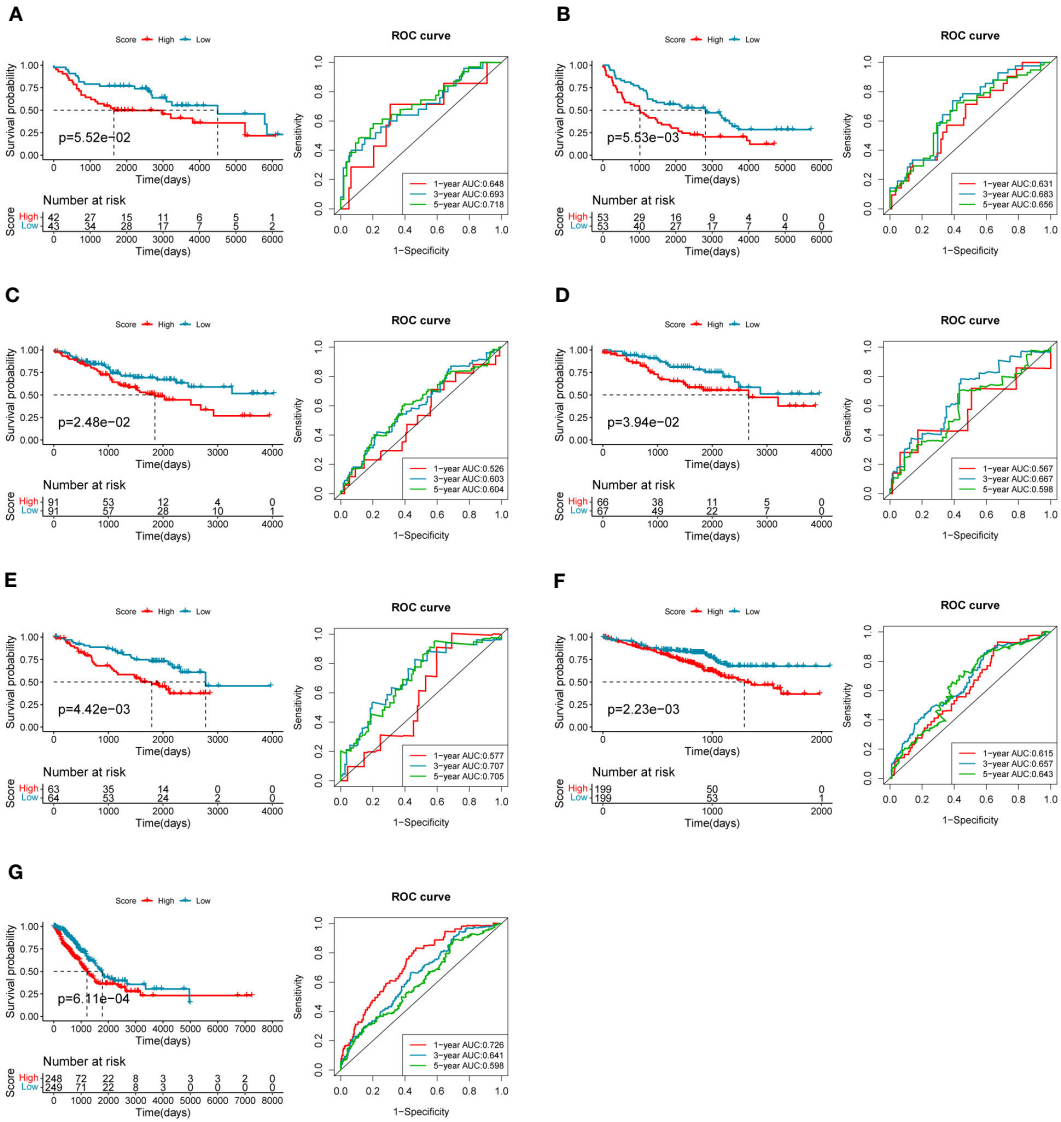
The predictive efficacy of the prognostic model in the external validation cohorts: **(A)** GSE30219 dataset, **(B)** GSE37745 dataset, **(C)** GSE41271 dataset, **(D)** GSE42127 dataset, **(E)** GSE50081 dataset, **(F)** GSE72094 dataset, **(G)** TCGA-LUAD dataset. On the left of each panel is the overall survival curve of patients in high and low-risk score groups. The abscissa axis shows survival time while the ordinate axis shows survival probability. Blue represents patients with low-risk scores while red represents patients with high-risk scores. The grouping status of the patients is indicated at the bottom of the chart. On the right of each panel is the ROC curve for predicting the 1-, 3-, and 5-year survival of LUAD patients according to the risk score. The abscissa axis represents specificity and the vertical axis represents sensitivity. Different colors represent different predictive times.

**Figure 5:**
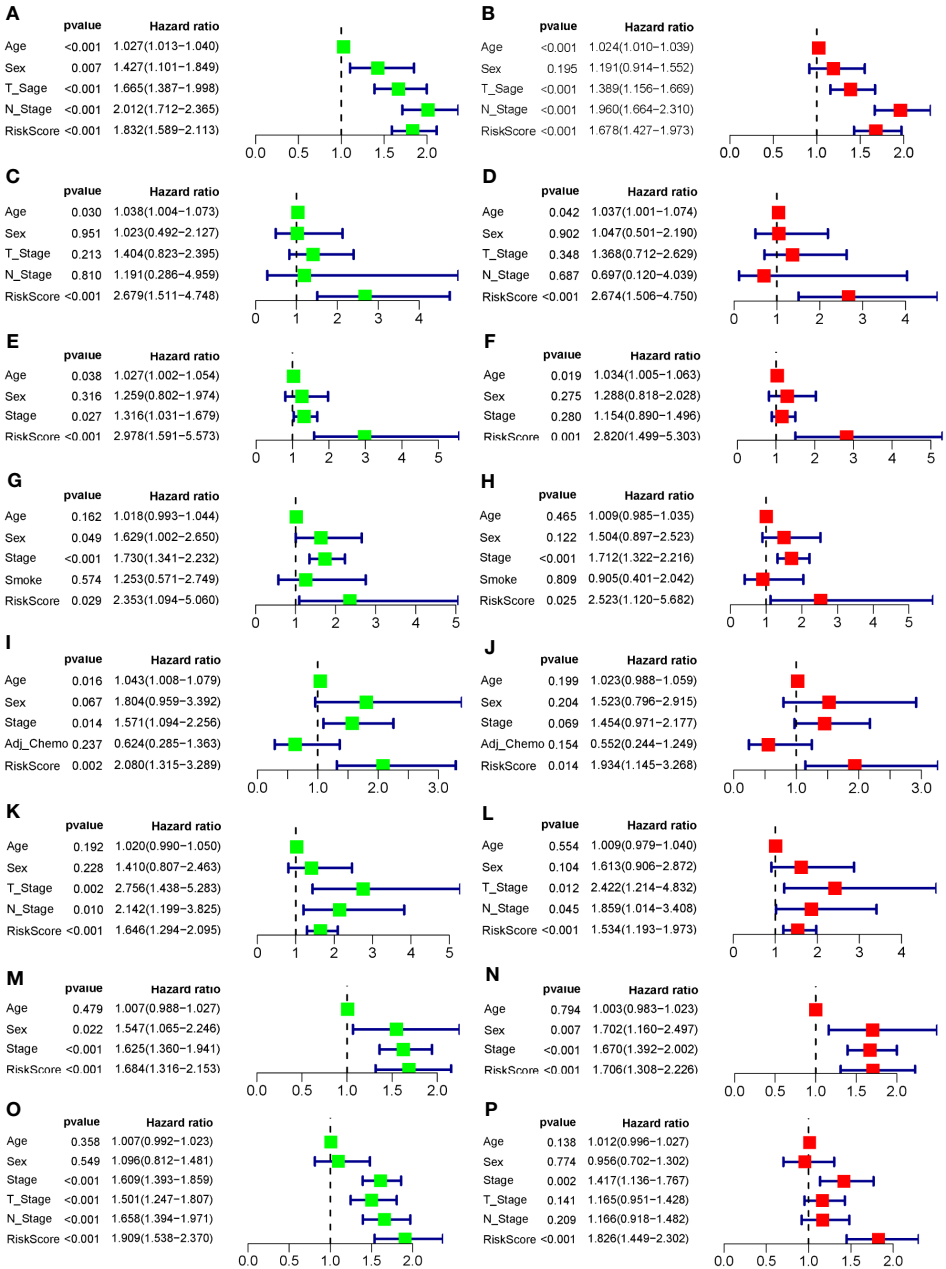
Forest plots of the univariate and multivariate Cox regression analyses for the prognostic model in the training and validation cohorts. **(A)** Univariate Cox regression analysis for the training cohort GSE68465. **(B)** Multivariate Cox regression analysis for the training cohort GSE68465. **(C)** Univariate Cox regression analysis for the validation cohort GSE30219. **(D)** Multivariate Cox regression analysis for the validation cohort GSE30219. **(E)** Univariate Cox regression analysis for the validation cohort GSE37745. **(F)** Multivariate Cox regression analysis for the validation cohort GSE37745. **(G)** Univariate Cox regression analysis for the validation cohort GSE41271. **(H)** Multivariate Cox regression analysis for the validation cohort GSE41271. **(I)** Univariate Cox regression analysis for the validation cohort GSE42127. **(J)** Multivariate Cox regression analysis for the validation cohort GSE42127. **(K)** Univariate Cox regression analysis for the validation cohort GSE50081. **(L)** Multivariate Cox regression analysis for the validation cohort GSE50081. **(M)** Univariate Cox regression analysis for the validation cohort GSE72094. **(N)** Multivariate Cox regression analysis for the validation cohort GSE72094. **(O)** Univariate Cox regression analysis for the validation cohort TCGA-LUAD. **(P)** Multivariate Cox regression analysis for the validation cohort TCGA-LUAD. The left column of each panel shows the *p*-value and hazard ratio of the factors, including risk score, and the right column shows the corresponding forest plot.

To evaluate the robustness and generalizability of the 10-gene COMAR prognostic model, several external independent datasets, including GSE30219, GSE37745, GSE41271, GSE42127, GSE50081, GSE72094, and TCGA-LUAD, were used as the validation cohort for this model. In both validation cohorts, the patients in the low- and high-risk groups had significantly different prognoses, and the ROC curves all indicated high sensitivity and specificity (**Figures 4A–G**). Furthermore, univariate and multivariate Cox regression analyses were applied to evaluate whether the risk score model could act as an independent prognostic factor for LUAD. In both training and validation cohorts, the risk score was considered to be an independent prognostic factor among other clinical features such as age, sex, and tumor stage (**Figures 5A–P**). These results all indicated that the 10-gene coagulation-related risk score model had a better prognostic efficacy with high robustness and generalizability.

### 3.5 Relationship between the COMAR risk score and the tumor microenvironment

**Figure 6:**
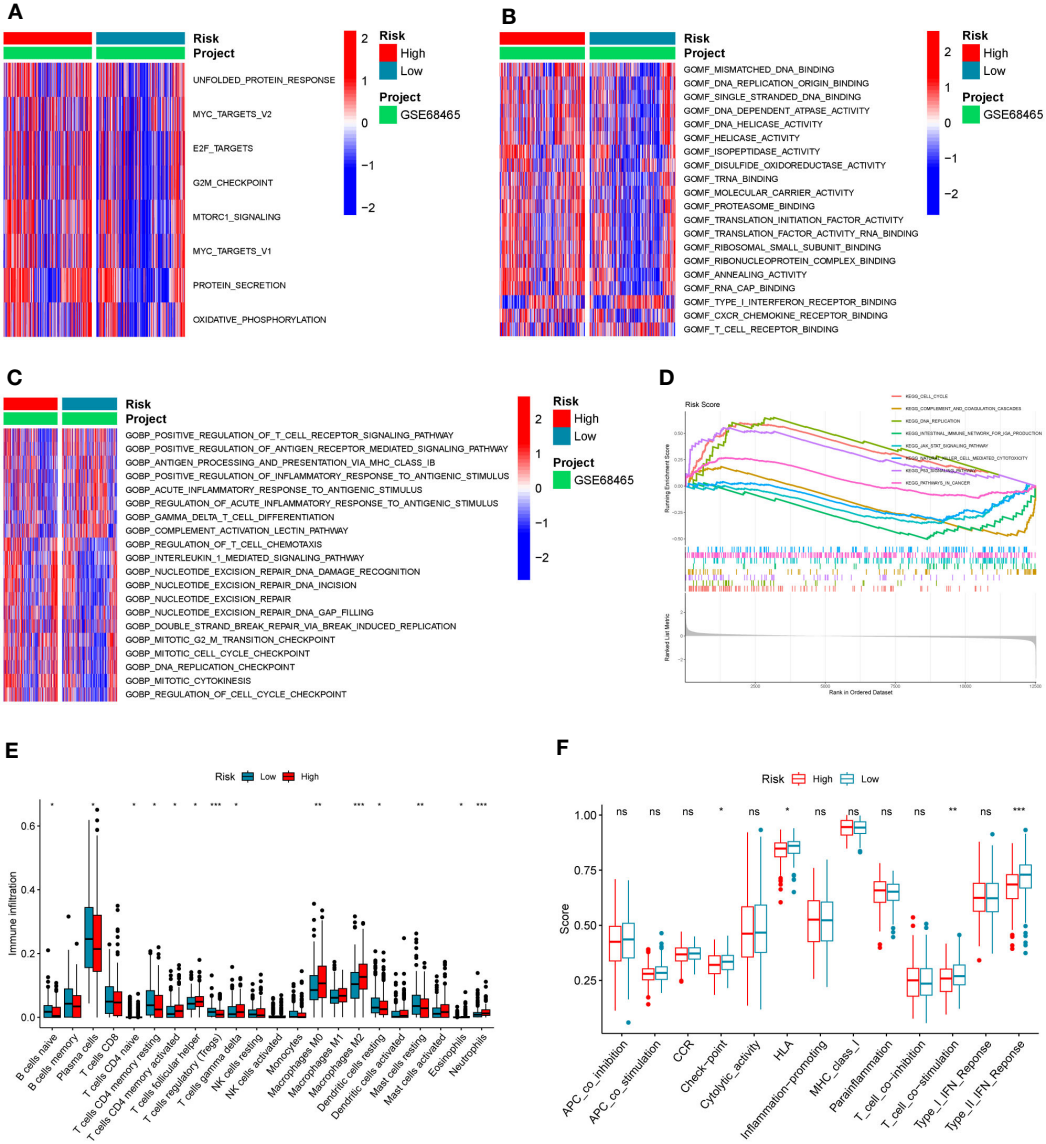
The association between the COMAR risk score and the TME characteristics. **(A–C)** GSVA enrichment analysis shows the differentially activated hallmarks **(A)**, GO_MFs **(B)**, and GO_BPs **(C)** between risk score low and high groups. The items of hallmarks, molecular functions, and biological processes are listed on the right. Red represents activation, while blue represents inhibition. **(D)** GSEA enrichment analysis shows the activated pathways in risk score high and low groups. The abscissa axis represents the ranked gene list according to their expression levels in two groups. The vertical axis represents the running enrichment score. Curves of different colors represent different pathways. The curves that have a high peak on the left side represent pathways that are enriched in the high-risk score group, while the curves that have a low peak on the right side represent pathways that are enriched in the low-risk score group. **(E)** Relative abundance of the 22 types of immune cells in risk score low and high groups. The abscissa axis represents the names of immune cells. The abscissa axis shows the immune cell types, and the vertical axis represents the infiltration fraction of each immune cell. **(F)** Score of functions in immune regulation in risk score low and high groups. The abscissa axis shows the items of immune functions, and the vertical axis shows the activation score of each immune function. "*p< 0.05; **p< 0.01; ***p< 0.001; ns, p> 0.05.

Different activations of hallmarks, GO_BPs, and GO_MFs in the GSE68465 dataset were investigated using the GSVA algorithm. Results indicated that some cancer hallmarks were much more enriched in the high-risk score group, such as MYC and MTOR-related pathways (**Figure 6A**; **Supplementary Table S6**). The high-risk score group had stronger molecular functions on DNA replication and transcription (such as DNA replication origin binding, helicase activity, and transcription initiation factor activity), while the low-risk score group exhibited greater molecular functions on immune activities (such as type I interferon receptor binding and T-cell receptor binding) (**Figure 6B**; **Supplementary Table S6**). Consistently, the immune biological pathways were mostly activated in the low-risk score group (such as positive regulation of T-cell receptor signaling pathway, positive regulation of antigen receptor-mediated signaling pathway, and positive regulation of inflammatory response to antigen stimulus), while pathways about DNA replication were activated in the high-risk group (such as DNA replication checkpoint and mitotic cell cycle checkpoint) (**Figure 6C**; **Supplementary Table S6**).

Similar to the results in the GO analyses, the KEGG GSEA indicated the high-risk score group was mostly enriched in the following pathways (DNA replication, cell cycle, and P53 signaling), while the low-risk score group was mostly enriched in the immune-related pathways (natural killer cell-mediated cytotoxicity, complement and coagulation cascades, and intestinal immune network for IgA production) (**Figure 6D**; **Supplementary Table S6**). To further explore the correlation between the risk score and tumor immune characteristics, the immune cell infiltration in these samples was investigated using the CIBERSORT algorithm. It was found that immune cell infiltration was overall higher in the low-risk score group than in the high-risk score group (such as naïve B cells, resting dendritic cells, naive CD4 T cells, resting memory CD4 T cells, and T follicular helper cells) (**Figure 6E**; **Supplementary Table S7**). However, the infiltration of macrophage M0 and M2 was significantly higher in the high-risk score group (**Figure 6E**; **Supplementary Table S7**). Moreover, some immune-related functions were much more activated in the low-risk score group, including HLA, T-cell co-stimulation, and type II IFN response (**Figure 6F**).

### 3.6 Predictive efficacy of the 10-gene COMAR model in immunotherapy

**Figure 7:**
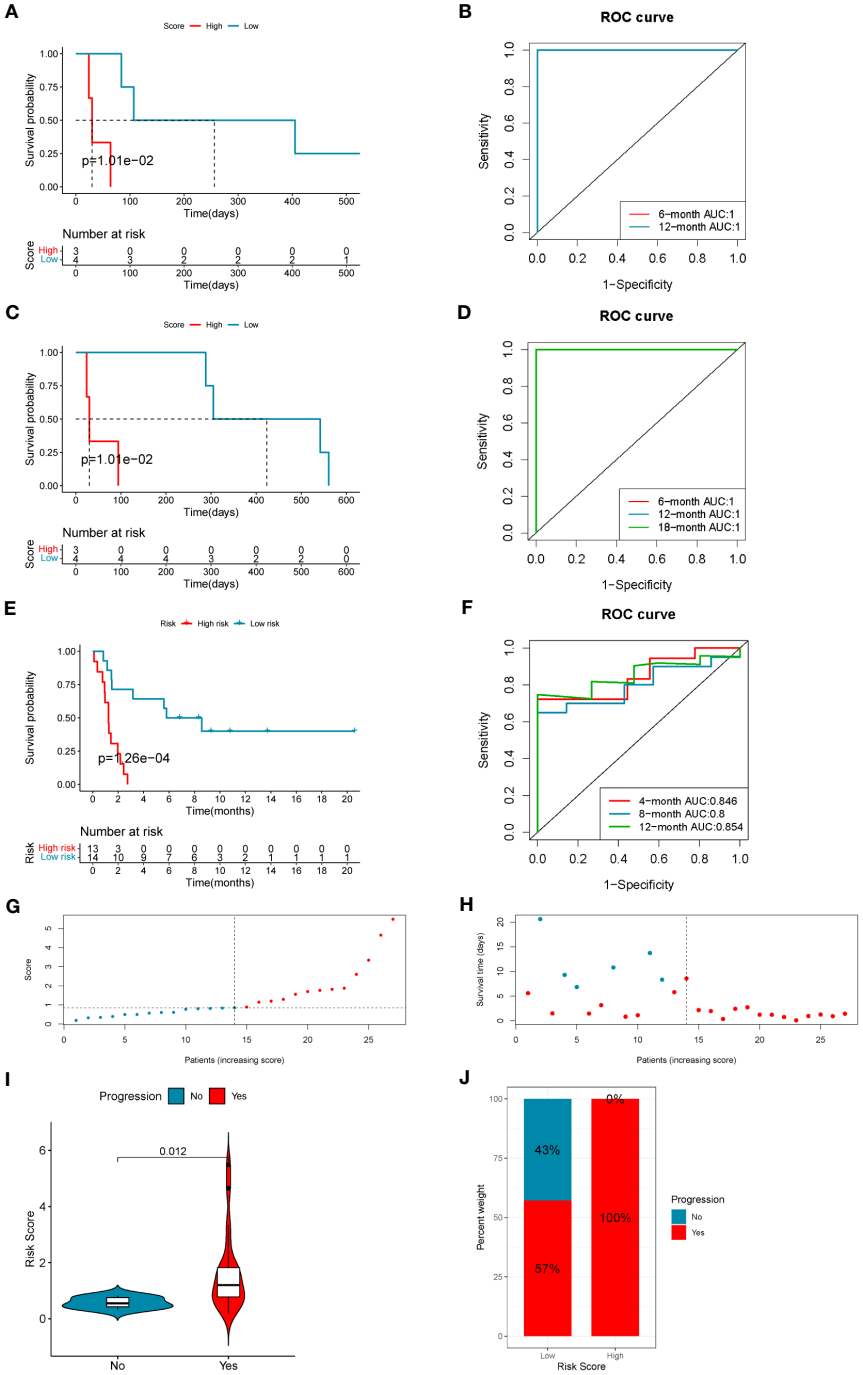
The 10-gene COMAR model predicts the immunotherapeutic outcomes of patients with LUAD. **(A)** The progression-free survival curve of patients with high and low-risk scores in the anti-PD-1 cohort GSE126044. **(B)** The ROC curve for predicting the 6- and 12-month progression-free survival of patients in the GSE126044 cohort. **(C)** The overall survival curve of patients with high and low-risk scores in the GSE126044 cohort. **(D)** The ROC curve for predicting the 6- and 12-month overall survival of patients in the GSE126044 cohort. **(E)** The progression-free survival curve of patients with high- and low-risk scores in the anti-PD-1/PD-L1 cohort GSE135222. **(F)** The ROC curve for predicting the 4-, 8-, and 12-month progression-free survival of patients in the GSE135222 cohort. For the survival charts, the abscissa axis shows survival time, while the ordinate axis shows survival probability. Blue represents patients with low-risk scores, while red represents high-risk scores. The grouping status of the patients is indicated at the bottom of the chart. For the ROC curves, the abscissa axis represents specificity, and the vertical axis represents sensitivity. Different colors represent different predictive times. **(G)** The risk score distributions of the patients in the GSE135222 cohort. **(H)** The survival status of the patients in the GSE135222 cohort. **(I)** Violin plot showing the risk score of patients with progression or no progression after anti-PD-1/PD-L1 blockade immunotherapy in the GSE135222 cohort. **(J)** The proportion of patients with progression or no progression after immunotherapy in low- and high-risk score groups in the GSE135222 cohort.

The risk scores of LUAD patients treated with anti-PD1/PD-L1 blockade in the GSE126044 and GSE135222 datasets were calculated using the risk score model. In the GSE126044 cohort, it was found that patients in the low-risk score group had significantly better progression-free survival (PFS) and overall survival (OS) versus high-risk score group (**Figures 7A, C**). Surprisingly, the corresponding ROC curves indicated that the AUCs at 6 months, 12 months, and 18 months were all 1 (**Figures 7B, D**). Similar results could also be found in the GSE135222 cohort. Patients in the low-risk score group had a remarkable advantage in prognosis (**Figure 7E**), and the AUCs of patients at 4 months, 8 months, and 12 months were 0.846, 0.8, and 0.854, respectively (**Figure 7F**). The risk score distributions and the survival status of the patients in the GSE135222 cohort are provided in **Figures 7G, H**. Moreover, patients who experienced progression of LUAD after anti-PD1/PD-L1 immunotherapy were found to have a higher risk score (**Figure 7I**), and they were all in the low-risk score group (**Figure 7J**). These results indicated that the 10-gene coagulation and macrophage-related model had a strong predictive efficacy for patients’ prognosis with anti-PD1/PD-L1 immunotherapy.

### 3.7 Validation of the bioinformatic analytical results through the patient specimens and cancer cell lines

**Figure 8:**
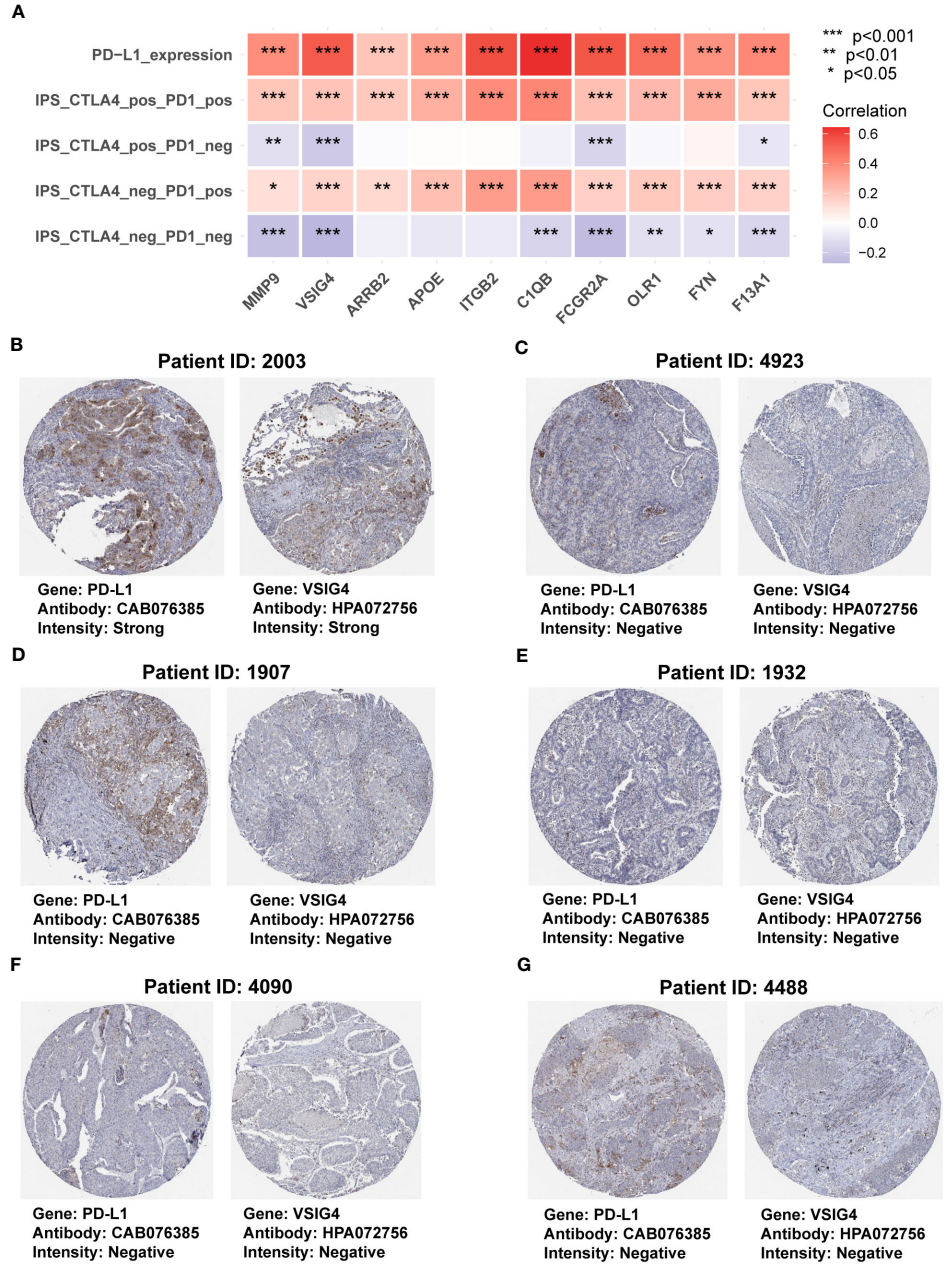
The correlation between genes in the COMAR model and anti-PD-1/PD-L1 immunotherapy. **(A)** Correlation analysis between the 10 genes involved in the COMAR model, PD-L1 expression, and IPS in immunotherapy. **(B-G)** Immunohistochemical staining images of the specimens of patient ID 2003 **(B)**, 4923 **(C)**, 1907 **(D)**, 1932 **(E)**, 4090 **(F)**, and 4488 **(G)**. Images were downloaded from the Human Protein Atlas (HPA) database. Gene names, antibodies, and staining intensity are listed at the bottom of each image. IPS, immunophenoscore. *p< 0.05; **p< 0.01; ***p< 0.001.

To further investigate the functions of the genes in the COMAR model in immunotherapy, first we made a correlation analysis in the TCGA-LUAD dataset and found the expression levels of all the genes in the COMAR model were positively correlated with PD-L1 expression level and the immunophenoscore (IPS) with anti-PD1+CTLA4 or anti-PD1 along immunotherapy (**Figure 8A**). Next, we analyzed the protein expression levels of the COMAR genes and PD-L1 in the HPA database, and the immunochemical images of VSIG4 and PD-L1 of six patients stained using the same antibody for each gene were obtained.

It was found that patient 2003 with the strong staining intensity of PD-L1 could also be found with the strong staining intensity of VSIG4 in the specimens. Furthermore, the specimens of the other five patients with negative staining of PD-L1 were consistent with the negative VSIG4 staining results (**Figures 8B–G**). This indicated that the COMAR genes were positively correlated with PD-L1 expression at the proteinic level. We also explored the correlation between the 10 COMAR genes and immune checkpoint genes in LUAD cell lines using the data from the Cancer Cell Line Encyclopedia (CCLE) database (29). The results indicated that the expression levels of some COMAR genes, like ITGB2, were positively correlated with multiple immune checkpoints (**Supplementary Figure S6A**), which was consistent with the results in the patient specimens. Moreover, we obtained the immunofluorescent staining image of the VSIG4 gene in LUAD cell line A-549 from the HPA database and found that VSIG4 is mainly located in the plasma membrane and cytosol of cancer cell line A-549 (**Supplementary Figure S6B**).

## 4 Discussion

It had been extensively reported that lung cancer, especially for LUAD, could frequently cause coagulation aberration and even venous thromboembolism, which was a major cause of cancer-related deaths (25–27, 30–32). It has been proved that the TME plays a significant role in tumor progression and therapy. As an essential component of the TME, TAMs have been the focus of several studies. TAMs could facilitate the progression of most types of cancer, including LUAD, through promoting angiogenesis, suppression of specific immunity, and cancer growth and metastasis (7, 8, 11–16). TAMs could also be applied as the therapeutic target for cancers, and the ways include depleting them, reverting TAM polarization, checkpoint blockade, strategies to reshape and activate TAMs, metabolic approaches, and macrophage cell therapies (7, 8). TAMs played an important role in coagulation, which was closely related to cancer development. For example, TAMs could produce factor X (FX) and activate the cell-autonomous FXa-PAR2 signaling in the TME, which led to tumor immune evasion and a poor prognosis (19, 20). Some other coagulation-associated factors could also strengthen the tumor-promoting effects of TAMs (21–23). Therefore, targeting the coagulation-related factors might effectively dampen the tumor-promoting functions of TAMs and boost the efficacy of cancer therapy. There have been some studies showing that targeting coagulation signaling could inhibit or reprogram TAMs and improve immunotherapy (19, 20). However, the regulatory mechanisms between coagulation and TAMs in tumor development still need to be further studied, and more biomarkers related to the coagulation process and TAM functions that could be used for cancer therapeutic targets and prognostic prediction should be explored.

In this study, we acquired the coagulation-related genes from the coagulation pathways provided by MSigDB and KEGG databases (**Table 1**; **Supplementary Table S1**). Then, we found high macrophage M2 content in the tumor was associated with a worse prognosis in LUAD patients while macrophage M1 was not (**Supplementary Figures S2A, B**), so the macrophage M2-related genes were identified using the WGCNA method in the bulk RNA-seq data (**Supplementary Figures S2C–G**; **Supplementary Table S2**). Single-cell sequencing is an advanced technology that gives us an unprecedented opportunity to dissect cellular heterogeneity in various biological contexts by analyzing transcriptomic profiles of thousands to millions of cells simultaneously (33–36). Through analyzing the scRNA-seq data, we annotated all the cell types and characterized the TAM marker genes in the LUAD scRNA-seq data (**Supplementary Figure S3C**; **Supplementary Table S3**) (28). Finally, we adopted the intersectional genes of the three groups of genes and obtained 33 genes that are closely related to the coagulation process and TAM infiltration for further analyses (**Figure 2A**; **Supplementary Table S4**). Those genes were named COMAR genes.

Subsequently, we performed K-M survival analysis for those 33 COMAR genes and found that 19 genes were associated with the prognosis (**Supplementary Figure S6**). Based on the 19 genes, we constructed a prognostic model including 10 genes, which was effective and proved robust in predicting patients’ prognosis (**Figures 3** **–** **5**). Among the 10 genes, ARRB2 was reported to be a tumor suppressor and could inhibit the progression of various kinds of cancer, including lung cancer (37–41). In our study, ARRB2 was found to be a protective factor for prognosis, which was consistent with the previous studies (**Figure 3B**). Moreover, it was found that ARRB2 was significantly downregulated in tumor versus normal tissues and presented with a higher frequency of CNV deletion (**Figures 2B, D**). Thus, we speculated that the expression of ARRB2 might be regulated by CNV in LUAD.

F13A1 was an important coagulation-related gene encoding factor XIII subunit A (FXIII-A), which was a transglutaminase involved in hemostasis, wound healing, tumor growth, and apoptosis (42). It was reported that F13A1 was a risky factor for the prognosis of patients with several types of cancer (43–45), which was consistent with our study (**Figure 3D**). Though F13A1 had a high frequency of CNV amplification (**Figure 2B**), it was downregulated in LUAD tumor tissues (**Figure 2D**). Considering its high mutation rate (**Figure 2C**), we speculated that mutational inactivation might be the reason for the low expression of F13A1.

C1QB was a risky factor for patients with some cancers, including NSCLC, according to previous studies (46–48), which was also consistent with our analyses (**Figure 2C**). CIQB might affect prognosis by regulating the TME because a study found that intrahepatic cholangiocarcinoma (ICC) with APOE^+^C1QB^+^ subtype of macrophage infiltration was associated with the chronic inflammation subtype of ICC and poor prognosis (46).

The FCGR2A gene encodes a member of the immunoglobulin Fc receptor gene family (49). Previous studies mainly focused on the polymorphisms of this gene that could influence the clinical outcomes of monoclonal antibody treatment in cancers like breast cancer (50), colorectal cancer (51, 52), and neuroblastoma (53). Only limited reports pointed out that high expression of FCGR2A was associated with a poor prognosis for cancer patients (49, 54). In our study, we also found that high expression of FCGR2A was associated with shorter survival in LUAD (**Figure 3E**). Moreover, FCGR2A, presented with a higher rate of CNV amplification, was downregulated in LUAD tumor tissues (**Figures 2B, D**), which was rarely reported. Thus, it needed to be further explored, and the regulatory mechanisms of its expression that were not consistent with CNV amplification also needed to be figured out.

FYN was a nonreceptor tyrosine kinase (RTK) member of the Src family kinase (SFK) (55). It was reported that FYN promoted tumor progression in glioma (56), melanoma (57), colon cancer (58), gastric cancer (59), and pancreatic cancer through various mechanisms (60). Furthermore, FYN was found to suppress LUAD by downregulating PI3K/AKT and inhibiting the epithelial-to-mesenchymal transition (61). This might partially account for the result of our study that FYN was a protective factor for the prognosis (**Figure 3F**).

ITGB2 participated in the YAP-induced cancer cell invasion by activating leukocyte-specific integrin β2 expression (62) and the myxofibrosarcoma aggressiveness conferred by SKP2 amplification (63). High expression in cancer-associated fibroblast (CAF) could promote oral squamous cell carcinoma proliferation by regulating PI3K/AKT/mTOR pathways to enhance glycolysis activity in CAFs (64). Moreover, high expression of ITGB2 was also reported to be correlated with poor prognosis in some cancers (65, 66). However, ITGB2 presented with opposite functions in NSCLC. It inhibited the proliferation and metastasis of NSCLC cells through suppressing EMT. Furthermore, low expression of it was associated with inferior prognosis in NSCLC (67), which was validated in an independent dataset, GSE68465 (**Figure 3G**).

MMP9 can degrade various components of the extracellular matrix to promote cancer cell invasion and liberate ligands for growth factor receptors from the extracellular matrix. It has been reported to play an important role in tumor-induced VEGF-dependent angiogenesis and prepping organs for the formation of distant metastases depending upon VEGFR-1 (68). Furthermore, it not only induced metastasis to the lung but was also involved in lung cancer invasion through multiple mechanisms (69). Consistent with previous reports, we found that MMP9 was also a risky factor for the prognosis of LUAD (**Figure 3H**).

The OLR1 gene encodes the LOX-1 receptor protein, which could facilitate the progression and metastasis of several cancers (70). OLR1 could also promote lung metastases of osteosarcomas through regulating the EMT (71). Similar to the above, OLR1 was also an unfavorable risky factor for the prognosis (**Figure 3I**). Its high frequency of CNV deletion might be the reason for its low expression in LUAD (**Figures 2B, D**).

VSIG4 was a multifunctional cell surface protein and presented as an immune checkpoint regulator, which suppressed T lymphocyte function and promoted cancer development and progression (72). In NSCLC tissues, VSIG4 could only be found expressed in macrophages, and the VSIG4^+^ macrophages infiltrating the tumor tissues could facilitate tumor growth by inhibiting T-cell proliferation and cytokine production (73). This might mechanically explain why the high expression of VSIG4 was related to the poor prognosis of LUAD (**Figure 3J**).

Different from previous reports, APOE was found to be a protective factor for the prognosis of LUAD (**Figure 3A**), while it was reported to promote cancer proliferation and migration and contribute to an aggressive clinical course in patients with LUAD (74). When APOE was knocked out, lung tumor development and metastasis were suppressed via increasing TREM-1-dependent antitumor activity of NK cells (75). In general, most of the genes involved in the COMAR prognostic signature were limitedly researched for their roles in LUAD. The regulatory mechanisms of coagulation aberrancy and TAM functions and the cross-talk relationships between them still need to be further studied.

For the correlation analysis between the COMAR risk score and the TME, patients in the low-risk score group were found to participate in much more activated immune-related biological pathways versus patients in the high-risk score group. These immune-related biological pathways might suppress tumor progression and contribute to a better prognosis for the low-risk group. In the GO_MF analysis, we found that the molecular functions of T-cell receptor (TCR) and type I interferon (IFN) receptor binding were enhanced in the low-risk score group (**Figure 6B**). For T cells, antitumor reactivity was defined by their unique TCRs (76), and high TCR abundance was associated with a better prognosis (77). Type I IFNs play a major role in the natural and therapy-induced immunological control of many malignancies, including lung cancer (78). The GO_BP analysis also indicated that positive regulation of the TCR pathway was activated in the low-risk score group. In addition, the gamma-delta T-cell differentiation was also found to be activated in the low-risk score group (**Figure 6C**). Gamma-delta T cells had antitumor functions in the TME and a high content of Vδ1 T cells; Vδ1 T cells were reported to be a subtype of gamma-delta T cells and were associated with superior prognosis and response to anti-PD-1 immunotherapy (79).

The GSEA of the KEGG pathway also indicated that several immune-associated pathways were enriched in the low-risk score group, including natural killer cell-mediated cytotoxicity (**Figure 6D**). Natural killer (NK) cells were cytotoxic lymphocytes of the innate immune system that were capable of killing viral infected and/or cancerous cells (80); when NK cells were commonly reduced in human tumors, immune surveillance escape would happen (81). The CIBERSORT analysis indicated that samples in the low-risk score group were infiltrated with a higher fraction of B cells, plasma cells, and CD4 cells, but less macrophage M2 (**Figure 6E**). B cells, plasma cells, and CD4 cells had been proven to play significant roles in promoting antitumor immunity and better clinical outcomes in the ICB immunotherapy (82–84), while macrophage M2 was associated with NSCLC progression, antitumoral immunosuppression, and resistance to anti-PD-1 immunotherapy (12, 15, 85, 86).

The results of the subsequent analyses in the LUAD immunotherapeutic cohorts corresponded to those of the TME analyses. Patients in the low-risk score group had significantly longer survival times and lower progression rates after accepting anti-PD-1 immunotherapy (**Figure 7**). The AUC values were pretty high, especially in the GSE126044 cohort (the AUC value = 1), which indicated the high sensitivity and specificity of this prognostic model (**Figures 7B, D, F**). Immune surveillance escape occurred by hijacking the corresponding inhibitory pathways via overexpressed checkpoint genes such as PD-L1 and CD47; thus, phagocytosis checkpoints have emerged as essential checkpoints for cancer immunotherapy (87). In the correlation analysis, we found that the 10 COMAR genes were positively correlated with immune checkpoint expression, such as PD-L1 and the IPS with anti-PD1 plus anti-CTLA4 or anti-PD1 along immunotherapy in both patient specimens and LUAD cell lines (**Figure 8**; **Supplementary Figure S6A**). Most of the genes in the COMAR model have also been reported to be positively correlated with PD-L1 expression and respond to ICB immunotherapy in multiple cancers (47, 88–93), which was consistent with the results of our study. These suggested that the 10 COMAR genes might serve as potential targets for ICB immunotherapy.

Certainly, there were also some limitations in our study. First, our study was mainly based on bioinformatic analyses of public datasets. Biological and molecular experiments *in vitro* and/or *in vivo* were needed to further explore the relevant mechanisms of the key COMAR genes. Second, due to our retrospective study, bias might be inevitable, and prospective experiments were needed for further validation. These limitations were also the focus of our future research. Our research had significant potential for future clinical guidance. First, the expression levels of key COMAR genes in LUAD could be examined before ICB immunotherapy and then applied for screening of immunotherapy patients. Second, researchers could explore the therapeutic target potential of these genes, which could be adopted for the development of targeted drugs.

In brief, the coagulation process and macrophage infiltration are two important factors that are usually aberrant in LUAD. They have cross-talk impacts on each other mutually and contribute to the concerto in regulating LUAD development. Based on the coagulation-related genes and the M2-TAM marker genes, a scoring model containing 10 prognostic genes (APOE, ARRB2, C1QB, F13A1, FCGR2A, FYN, ITGB2, MMP9, OLR1, and VSIG4) was constructed. This prognostic signature is super efficacious in predicting the prognosis and ICB immunotherapeutic outcomes of patients with LUAD, which provides potential biomarkers for LUAD treatment and prognostic prediction.

## Data availability statement

The original contributions presented in the study are included in the article/**Supplementary Material**. Further inquiries can be directed to the corresponding author.

## Ethics statement

Ethical approval was not required for the study involving humans in accordance with the local legislation and institutional requirements. Written informed consent to participate in this study was not required from the participants or the participants’ legal guardians/next of kin in accordance with the national legislation and the institutional requirements.

## Author contributions

ZLi: Data curation, Formal analysis, Investigation, Methodology, Writing – original draft. ZYi: Formal analysis, Data curation, Writing – original draft. ZLu: Data curation, Formal analysis, Writing – original draft. CZ: Data curation, Formal analysis, Writing – original draft. YW: Data curation, Formal analysis, Writing – review & editing. KZ: Data curation, Formal analysis, Writing – review & editing. FC: Data curation, Formal analysis, Writing – review & editing. ZYa: Data curation, Formal analysis, Writing – review & editing. YT: Conceptualization, Funding acquisition, Investigation, Methodology, Project administration, Supervision, Validation, Writing – review & editing.
