# Supplementary material for: Comprehensive analyses for the coagulation and macrophage-related genes to reveal their joint roles in the prognosis and immunotherapy of lung adenocarcinoma patients
Source: Front Immunol. 2023 Oct 31;14:1273422. doi: 10.3389/fimmu.2023.1273422 (PMC10644034; doi:10.3389/fimmu.2023.1273422)
Supplement: Supplementary file 1 [file DataSheet_1.pdf]

Supplementary Figures

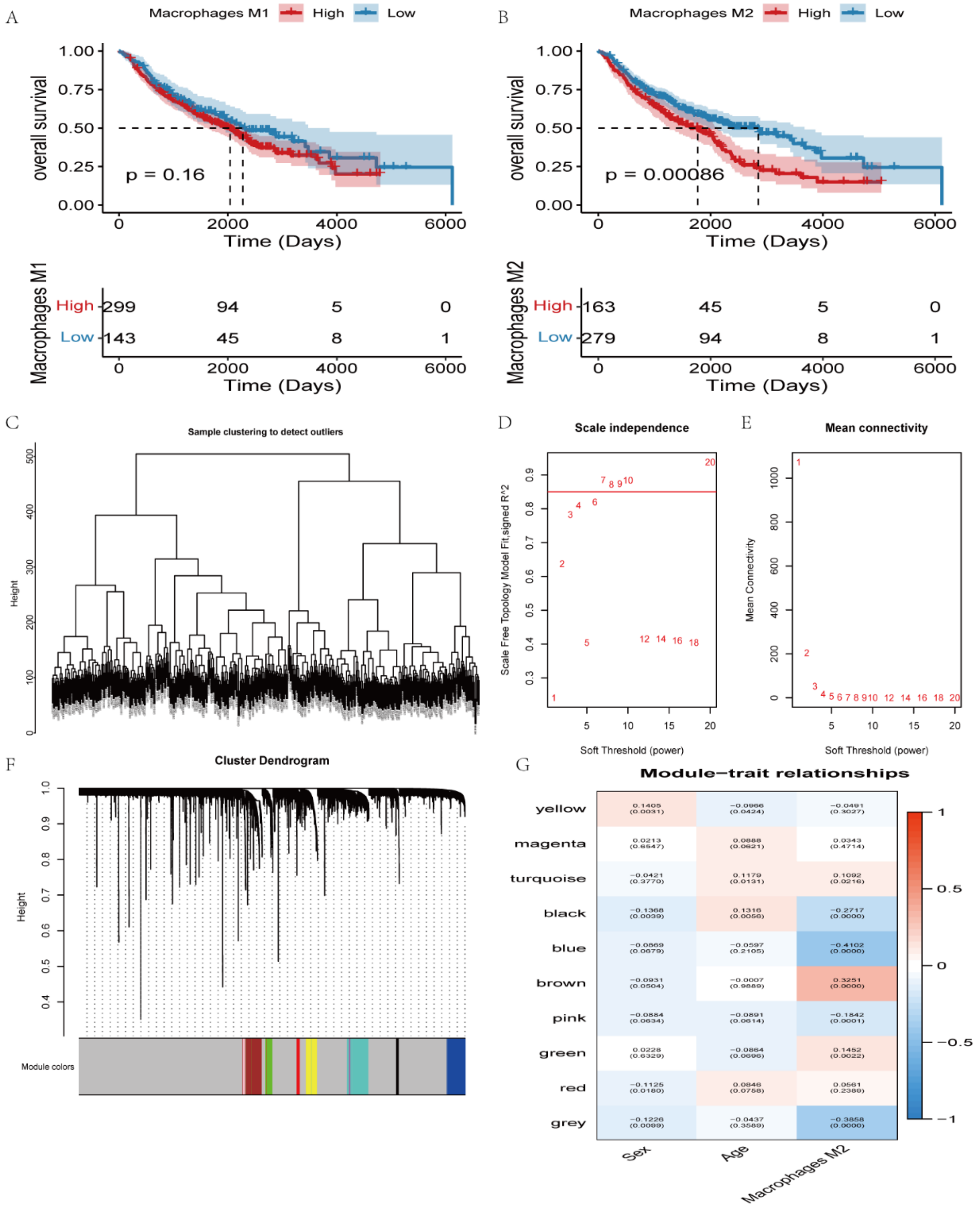

Supplementary Figure 1. Overall survival analysis for the subgroups that contain different abundance of macrophage. (A) Overall survival curve for the subgroups containing high and low abundance of macrophage M1. **(B)** Overall survival curve for the subgroups containing high and low

abundance of macrophage M2. The abscissa axis shows survival time while ordinate axis shows survival probability. Blue represents low macrophage abundance while red represents high macrophage abundance. The grouping status of the patients is indicated at the bottom of the chart. P < 0.05 in the Log-rank test was considered statistically significant. **(C)** Sample clustering in the WGCNA analysis without finding any outliers. **(D-E)** Detecting the optimal soft-thresholding power. When the power value is seven, the degree of independence was > 0.85 for the first time. **(F)** The cluster dendrogram of modular genes associated with macrophage M2 infiltration. Branches of the dendrogram correspond to the different gene modules. Each leaf on the dendrogram represents a gene. Each block marked by a color represents a module that contains a group of highly correlated genes. A total of 10 gene modules were identified. **(G)** Correlation between gene modules and clinical traits. The correlation coefficient and corresponding p-value are annotated in the blocks of the module-trait relationships heatmap. Red represents positive correlation and blue represents negative correlation. WGCNA, Weighted Gene Co-Expression Network Analysis.

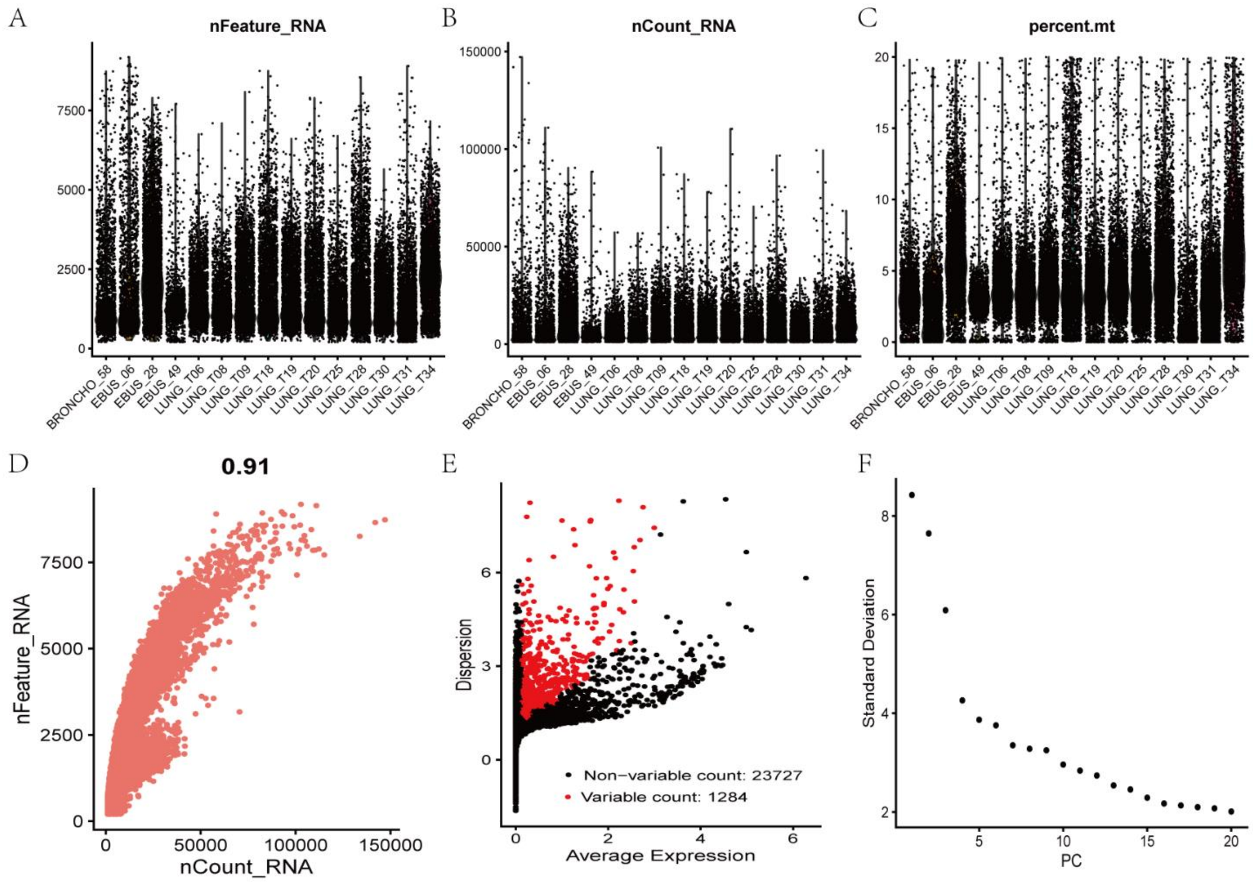

Supplementary Figure 2. The quality control for the scRNA-seq data. (A) The number of genes detected in each cell of each sample. The abscissa axis shows the names of the samples and the vertical axis shows the number of genes. Each black dot represents a cell. **(B)** The total number of Count in each cell of each sample. The abscissa axis shows the names of the samples and the vertical axis shows the number of Counts. Each black dot represents a cell. **(C)** The percentage of

mitochondrial genes in each cell of each sample. The abscissa axis shows the names of the samples and the vertical axis shows the percentage of mitochondrial genes. Each black dot represents a cell. **(D)** The correlation analysis shows the number of genes detected was positively correlated with the depth of sequencing. **(E)** The scatter plot of the top 3000 variable genes. **(F)** The top 20 PCs in the principal component analysis (PCA) for grouping the cells.

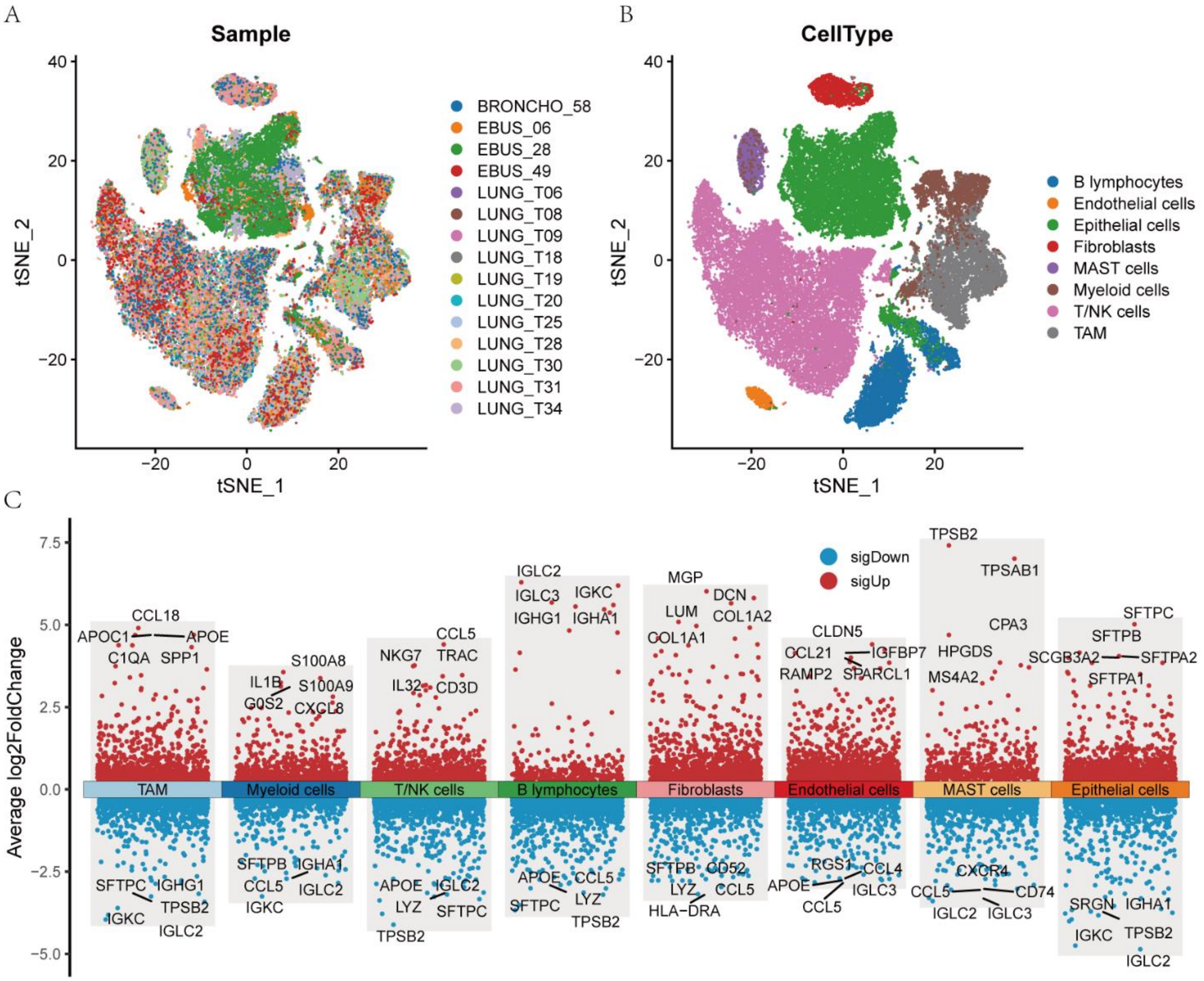

Supplementary Figure 3. The acquisition of the TAM marker genes. (A) The TSNE plot of the samples after removal of the batch effect in the harmony analysis. Different colors represent different samples. The names of the samples are annotated on the right of the plot. **(B)** The TSNE plot of the cells after removal of the batch effect in the harmony analysis. Different colors represent different cell types. The names of the cell types are annotated on the right of the plot. **(C)** The volcano plot showing the differentially expressed genes between different cell types. The red dots mean upregulated genes while blue dots mean downregulated genes.

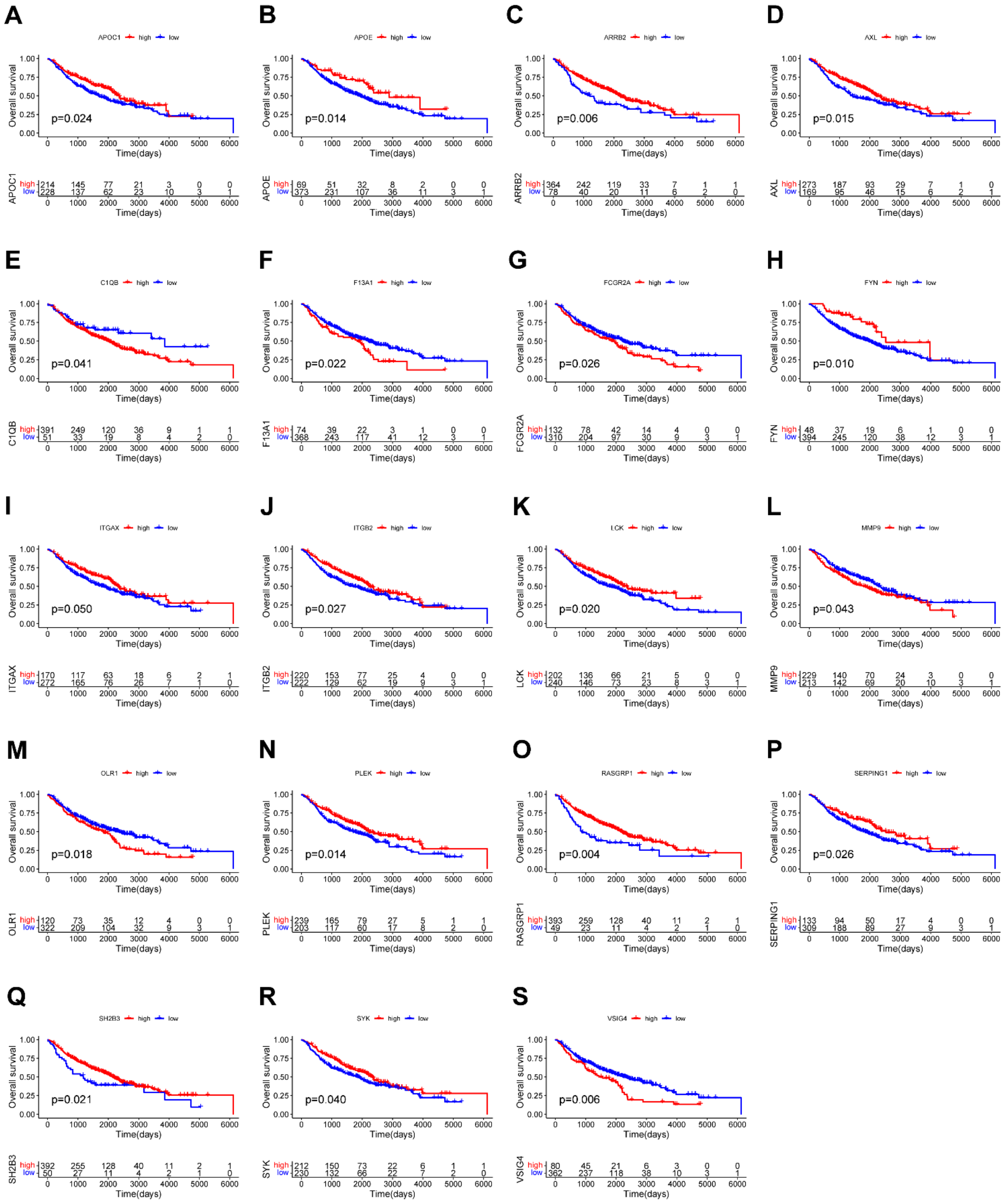

Supplementary Figure 4. The overall survival curves of the genes that are significantly related to prognosis among the 33 coagulation-associated genes: (A) APOC1, **(B)** APOE, **(C)** ARRB2, **(D)** AXL, **(E)** C1QB, **(F)** F13A1, **(G)** FCGR2A, **(H)** FYN, **(I)** ITGAX, **(J)** ITGB2, **(K)** LCK, **(L)** MMP9, **(M)** OLR1, **(N)** PLEK, **(O)** RASGRP1, **(P)** SERPING1, **(Q)** SH2B3, **(R)** SYK, **(S)**VSIG4. The abscissa axis shows survival time while ordinate axis shows survival probability. Blue color represents low expression while red color represents high expression. The grouping status of the patients is indicated at the bottom of the chart.

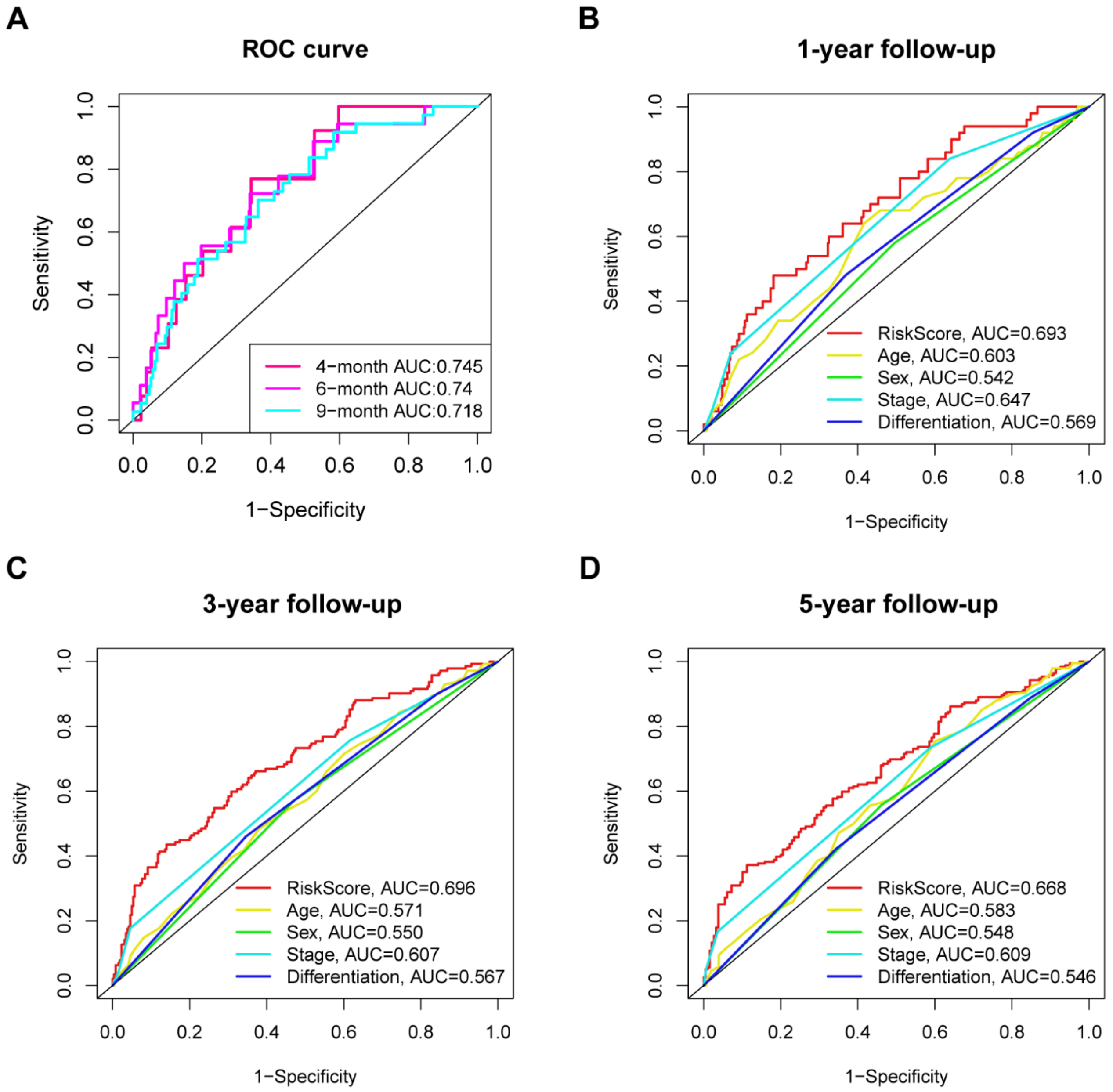

Supplementary Figure 5. (A) The ROC curve showing the AUCs of the COMAR model in predicting 4-month, 6-month and 9-month prognosis. **(B-D)** ROC analysis of the COMAR risk score, age, sex, tumor stage and differentiation status on the prognosis at 1-year **(B)**, 3-year **(C)** and 5-year **(D)** follow-up. The abscissa axis represents specificity and the vertical axis represents sensitivity.

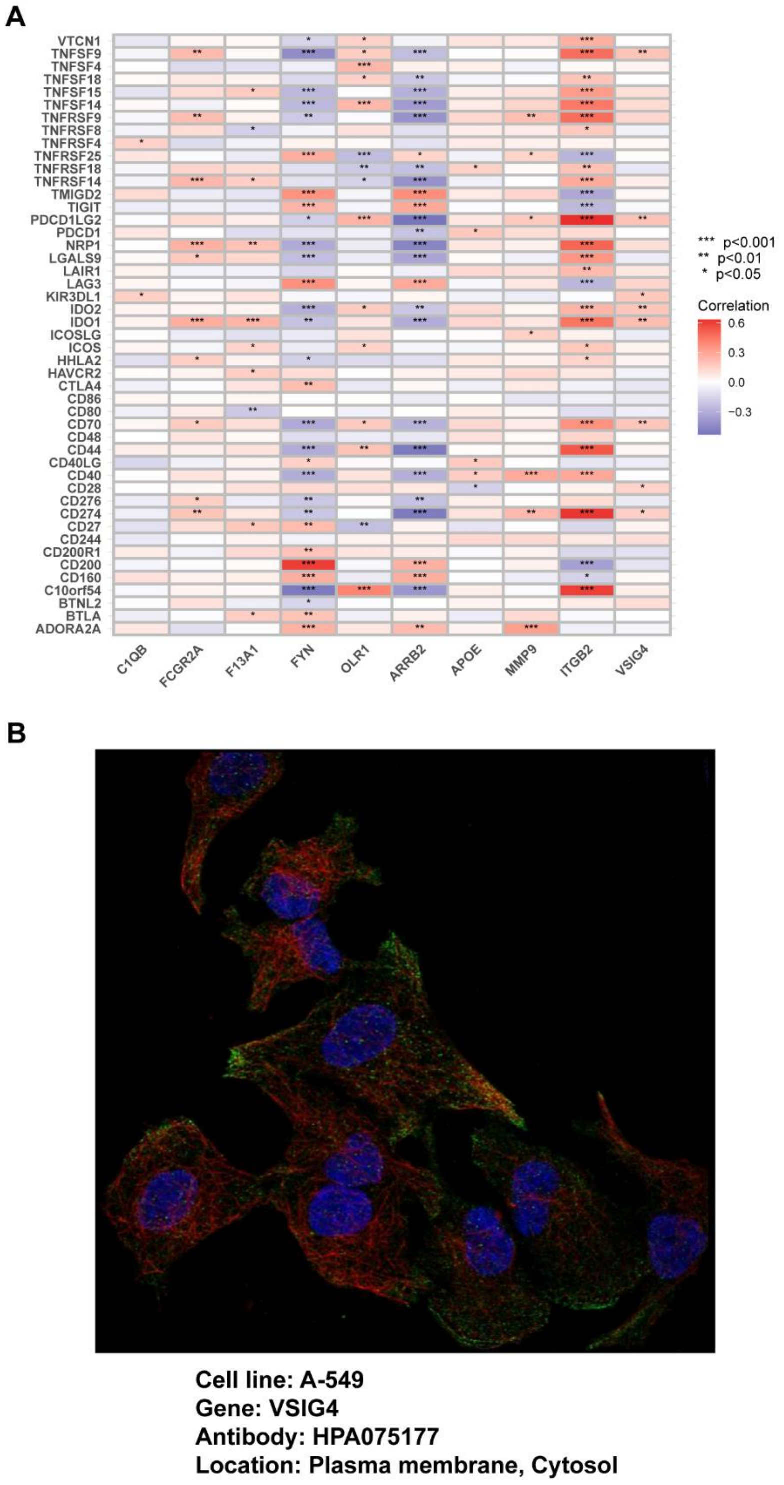

Supplementary Figure 6. The expression levels of the genes involved in the COMAR model in LUAD cancer cell lines. (A) Correlation analysis between the 10 COMAR genes and 47 immune checkpoint genes. **(B)** The immunofluorescent staining image of VSIG4 gene in LUAD cell line A-549 from the HPA database. Cell line, gene name, antibody and proteinic location were listed in the bottom of the image. The green color shows the target protein VSIG4, the red color shows the microtubes and the blue color shows the nucleus.
